# Aqueous Strippable Polymer Coating for Highly Efficient Primary Radioactive Uranium Decontamination with Versatility on Diversified Surface

**DOI:** 10.3390/polym14091656

**Published:** 2022-04-20

**Authors:** Yang Xue, Wuxinchen Yang, Renliang Yue, Yunfa Chen

**Affiliations:** 1 State Key Laboratory of Multi-Phase Complex Systems, Institute of Process Engineering, Chinese Academy of Sciences, Beijing 100190, China; yxue@ipe.ac.cn (Y.X.); ywxc666@163.com (W.Y.); rlyue@ipe.ac.cn (R.Y.); 2 Center of Materials Science and Optoelectronics Engineering, University of Chinese Academy of Sciences, Beijing 100049, China; 3 Center for Excellence in Regional Atmospheric Environment, Institute of Urban Environment, Chinese Academy of Sciences, Xiamen 361021, China

**Keywords:** strippable coating, surface radioactive uranium, primary decontamination efficiency, versatility, repeatability and mechanical properties

## Abstract

The decontamination of radioactive materials on the surfaces of nuclear facilities has generated large quantities of waste from the rapid development of the nuclear industry, posing a potential threat globally. Strippable coating has been employed for some time to remove radioactive contamination due to its high performance and removability, flexibility, and compatibility with various substrates. Herein, an aqueous strippable coating based on an adsorbent/polyvinyl alcohol (PVA) polymer was developed to remove radioactive uranium from stainless-steel surfaces that showed greater decontamination than that of DeconGel, with an efficiency of 87.2% for 5 g/L uranium and 95.5% for 22.5 g/L uranium, along with a high repeatability and better mechanical properties. Furthermore, the prepared coating was versatile and could be applied to a range of substrate surfaces (lacquered, aluminum, glass, plastic, and ceramic), with outstanding performance ranging from 79.2 to 95.4% for 1 g/L uranium. The prepared coating could also be applied through brushing or spraying to horizontal or vertical substrates. The exceptional performance could be due to the synergistic effect of the introduction of ethylene diamine tetra-acetic acid disodium salt (EDTA-2Na) as a chelating agent and the nano-adsorbent CaCO_3_/TiO_2_.

## 1. Introduction

Today, global energy requirements are increasing rapidly owing to the high-speed growth of industry and populations. Nuclear energy utilization, particularly uranium, has aroused extensive attention since the mid-20th century as a sustainable solution to the global energy crisis [1,2,3,4]. However, hazardous radioactive outputs, especially uranium, from nuclear power production have been unintentionally released into peripheral environments such as the surfaces and surroundings of nuclear facilities [5]. It is necessary to develop suitable radioactive decontamination technology to restore extensively polluted surrounding areas.

In comparison with the physical method with high-pressure water/acid solution washing [6], commonly used for rapid responses to large-scale decontamination, the strippable coating technique, involving brushing or spraying on a film-forming polymer coating to adsorb and decontaminate surfaces [7,8,9], has attracted the attention of the scientific community due to its outstanding advantages, including only producing solid waste without secondary waste liquid [2].

Recently, researchers have conducted increasing numbers of studies on strippable coatings that aim to provide a higher performance as well as eco-friendly, biodegradation, and self-embrittling characteristics [8,10,11,12]. Optimizing primary decontamination performance should focus on designing strippable coatings that reduce secondary solid waste pollution and protect human health. Yang et al. developed a new spray coating based on an adsorbent/polyvinyl alcohol (PVA)-borax complex that displayed a 137Cs removal efficiency of 56.966%, twice that of a commercial strippable coating (DeconGel, 27.248%), on porous cement surfaces due to its nano-clay adsorbent adjustment/screening among Prussian blue, bentonite, and sulfur-zeolite [8]. Pulpea et al. investigated and found differences in the effectiveness of decontaminating radioactive isotopes, employing eight complexing agents including iminodisuccinic acid (IDS), 2-phosphonobutane-1,2,4-tricarboxylic acid (PBTC), and EDTA with PVA-based biodegradable coating [9]. Furthermore, Toader confirmed a synergistic effect between nano-clay and a complexing agent that achieved up to three times the maximum decontamination of bentonite and EDTA using a PVA coating on a heavy metal surface [13]. The choice of both nano-clay and a chelating agent as adsorbents is essential for a high surface decontamination performance.

In addition to screening adsorbents for enhanced surface decontamination, the substrate species and radioactive elements should also be taken into consideration due to the quite different adhesion of different coatings to different substrates and their varied radioactive species adsorption capacities [14,15,16]. Pulpea systematically investigated differences in surface decontamination effectiveness based on the five most common contaminated areas, galvanized metal sheets (GMS), painted metal (PMe), ceramic tiles (CT), and plastic material (PMa), and four radioactive isotopes. The results showed a decontamination of over 95% for 137Cs (cesium; galvanized metal sheets, painted metal, ceramic tile and plastic material surfaces), over 90% for 60Co (cobalt) and 133Ba (barium; galvanized metal sheets, painted metal, ceramic tile and plastic material surfaces), and more than 72% for 241Am (americium) (painted metal, ceramic tile and plastic material surfaces: over 70% for 133Ba, maximum 41.87% for 241Am, and 43.19% for 60Co) [9]. Gruau also tested the ability of two commercial coatings produced by DeconGel, named DeconGelTM1101 and DeconGelTM1102, to decontaminate 137Cs and 60Co on five substratematerials. The quantitative results indicated that both the substrate and the radiative elements greatly influenced the percentages of contaminant removal [17]. These findings indicate an urgent need for a high-performance, versatile aqueous polymer strippable coating for primary radioactive uranium decontamination.

Of particular interest for surface contamination of nuclear facilities are gloveboxes, walls, and tools contaminated by the radioactive isotopes of uranium [5]. 137Cs, which is nonvolatile and tightly bound to the soil, and 60Co, a corrosion product, are common radiative markers used for measuring different coatings’ decontamination effectiveness [18,19,20]. Uranium decontamination waste contains low-level radioactive wastewater, and soil through vacuum membrane distillation and acid washing technologies are widely studied for uranium recovery [6,21,22,23]. However, conventional strippable coatings often are toxic, containing carcinogenic solvents and chelators and other materials such as ammonia; their application is unpleasant, and their development is still in its infancy [5]. Gray developed a smart coating containing PVA, glycerine, and diethylenetriaminepentaacetic acid (DTPA) together with an indicator that was responsive to uranium contamination [5]. Yin designed a polyaniline coating doped with a SO_4_^2−^/TiO^2^ solid superacid to replace HCL for greater uranium decontamination [24]. Wang investigated application dosages of a strippable coating containing acrylate emulsion and surfactant and found optimal decontamination of 92.26% with uranium dust on a concrete surface [25].

Considering the complexity of nuclear facilities, strippable coatings for practical application should also achieve certain structural and mechanical properties such as easily peeling off horizontal and vertical surfaces together with environmental friendliness [9,26,27,28].

Herein, polyvinyl alcohol (PVA), a nontoxic, water-soluble, completely biodegradable synthetic polymer, was chosen as the main component in the decontamination formulations due to its excellent film forming and adhesive properties [9]. Nano-fillers (CaCO_3_/TiO_2_) and chelating (EDTA) adsorbents were employed not only for their remarkable mechanical and thermal properties but especially for their outstanding ability to adsorb radioisotopes for optimal decontamination. Furthermore, film-forming auxiliary (modified chitosan and starch) was also used to obtain peeling coatings with proper mechanical properties. Normally, decontamination effectiveness will decrease markedly with increasing concentrations of radioactive elements. The aqueous PVA strippable coating in this study was first fabricated and evaluated for removal of polychlorinated biphenyls and uranium. The sample exhibited highly effective primary radioactive uranium decontamination, 87.2% for 5 g/L and 95.5% for 22.5 g/L uranium on stainless steel plates, twice the effectiveness of the commercial DeconGel sample; the sample also exhibited high repeatability, strength, and versatility with a range of substrate surfaces (lacquered, aluminum, glass, plastic and ceramic: 79.2 to 95.4% for 5 g/L). The sample also demonstrated potential for brushing or spraying on both horizontal and vertical substrates.

## 2. Materials and Methods

### 2.1. Materials

Polyethylene glycol (PEG-200, Coolaber, Beijing, China), modified starch (Fuchen Chemicals, Tianjin, China), chitosan (Aladdin), ethylene diamine tetra-acetic acid disodium salt (EDTA-2Na, Aladdin, Shanghai, China), nano calcium carbonate (CaCO_3_, Xianfeng NANO, Nanjing, China), nano titanium oxide (TiO_2_, Macklin, Shanghai, China), nitric acid (HNO_3_, Sinopharm Chemical, Beijing, China), sodium hydroxide (NaOH, Sinopharm Chemical, Beijing, China), poly(vinyl alcohol) (PVA, 99% hydrolysis degree, Macklin, Shanghai, China), uranium compounds (UO_2_(NO_3_)_2_·6H_2_O), polychlorinated biphenyls (Aroclor 1016, J&K Chemicals, Beijing, China), wipes (GhostWipe™, Charleston, SC, USA).

### 2.2. Methods

In this work, two aqueous polymeric coatings were prepared to test their decontamination performance with organic and radioactive contaminants. First, the PVA was dissolved in deionized water by stirring and heating at 90 °C to prepare a PVA aqueous solution. Then, the predispersed CaCO_3_ (or TiO_2_) solution was added into the PVA solution. After mixing for 30 min, EDTA-2Na aqueous solution (10 wt.%) was added dropwise and stirred for 40 min. Then, modified chitosan (or modified starch) was also added into the PVA solution. Finally, PEG-200 was added and stirred in for a few minutes. Notably, chitosan should be dissolved in 2% acetic acid solution at 60 °C before use. Blue pigment was also added after the solution had cooled to room temperature. The components of the two polymeric coatings are listed in Table 1.

### 2.3. Characterization

#### 2.3.1. Surface Decontamination of the Polychlorinated Biphenyl (PCB) Contamination Solutions

Decontamination of PCBs using gas chromatography-mass spectrometry (GC-MS, QP2010 Ultra, Shimadzu, Japan):

The aroclor 1016 contaminant was dissolved in DMSO (100 μL/1 mL), and 0.4 mL solution was brushed on aluminum plates (150 mm × 75 mm × 0.5 mm). After that, the prepared polymeric coating was poured onto the contaminated surface to dry for 24 h. Then, the dried films were peeled off, and the surfaces were swiped with wipes wetted with DMSO solvent (2 mL) according to ASTM E1728-03. The wipes were then immersed in sample tubes with 40 mL DMSO in each for 24 h to dissolve the collected PCBs. After that, the solvents were analyzed using GC-MS according to EPA SW-846. For contrast, a sample without polymeric coating was also prepared by swipe testing following the method above.

In addition, aroclor 1016 dissolved in DMSO solvent was prepared at different concentrations of DMSO to generate standard curves. Decontamination effectiveness was calculated as follows:

$$\mathrm{Decontamination}\;\mathrm{efficiency}\;\eta =\frac{A_{control}-A_{residual}}{A_{control}}\times 100\%$$

where $A_{control}$ was the contaminant concentration of the control wipe, and $A_{residual}$ was the contaminant concentration of the residual wipe.

#### 2.3.2. Surface Decontamination of the Radioactive Contamination Solution

Decontamination of uranium compounds using surface contamination testing:

The surface contamination tester measurements were taken using a surface contamination tester (CoMo170, Clover Technology Group. Inc., Beijing, China) according to GB/T14056.1-2008.

First, the sample surface was treated with sandpaper and immersed in the NaOH and HNO_3_ solutions, then washed and air dried to make uranium compounds adhere to the board more stably. After that, 1 g/L of a 10 mL uranium nitrate solution was painted on the stainless-steel plates (200 mm × 150 mm × 5 mm) and dried at room temperature. Then, the surface contamination monitor was used to test the surface contamination level of the sample before decontamination using the polymeric coating. Then, the polymeric coating was evenly painted on the surface of the contaminated sample to ensure that the contaminated area was completely covered, and the sample was dried at room temperature. The strippable film was peeled off, and the final surface contamination level was recorded; the average of every three test points was used in calculating the sample’s surface contamination level, including the initial and final values. Other substrates such as plastic plates (200 mm × 150 mm × 5 mm) were also used to test the decontamination effectiveness of the polymeric coating, calculated as follows:

$$\mathrm{Decontamination}\;\mathrm{Effectiveness}\;\eta =\frac{A_{0}-A_{1}}{A_{0}}\times 100\%$$

where *A*_0_ and *A*_1_ are the initial and final ^238^U activity on the surfaces, respectively, before and after treatment with the polymeric coating.

#### 2.3.3. Morphology of the Strippable Film

The surface morphology of the samples was observed using cold field emission scanning electron microscopy (FESEM) (SU8020, Hitachi, Tokyo, Japan) with an accelerating voltage of 20 kV.

#### 2.3.4. Mechanical Properties of the Strippable Film

Mechanical properties were measured using a Z010 material testing machine (Zwick/Roell, Ulm, Germany) at room temperature at the speed of 500 mm/min according to ASTM D412 and ASTM D624.

## 3. Results and Discussion

### 3.1. Decontamination Process of the Polymer Strippable Coating

**Figure 1:**
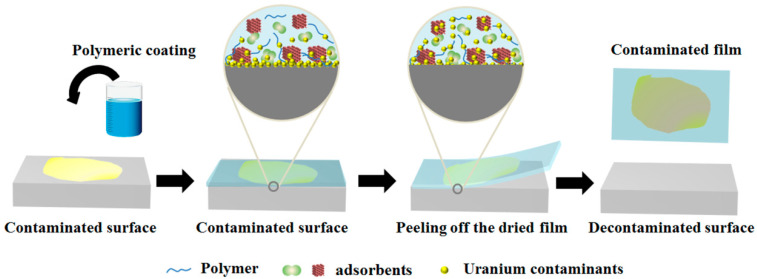
Schematic illustration of the decontamination process of polymeric coating.

The schematic representation of the whole decontamination process using an adsorbent/PVA-based strippable coating is displayed in Figure 1. Polymer solutions with proper viscosity containing adsorbents are poured onto a contaminated surface, then a viscous coating is generated and adheres to the uranium-contaminated surfaces to achieve uranium adsorption through fillers. The coating is allowed to dry for 12 h at room temperature and in an atmospheric environment to obtain the destination layer. The uranium contaminant was dissolved into this coating via interaction with the polymer molecule, nano-fillers, and chelating agent as the dry film formed.

Chitosan and starch are natural polymers with many functional groups such as hydroxyl, and the many hydroxyls in crosslinked modified chitosan/PVA and modified starch/PVA hybrid membranes might participate in decontamination through coordination with metal uranium ions [29]. Moreover, nano-fillers such as CaCO_3_ nanoparticles with high surface binding energy for their small particle size and ample surface atoms, have strong adsorption capacity with metal ions. The adsorption of uranium contaminants is mainly due to the complexation of the outer layer. That is, the atoms on the surface of the particles tend to coordinate with ions in water because they are unsaturated; thus, it is easy for these atoms to adsorb uranyl ions and quickly become stable, similar to the adsorption of uranyl ions on sodium rectorite [30]. In addition, the chelating agent EDTA-2Na absorbs uranyl ions to form EDTA-U chelated compounds to remove uranium contaminants [31,32]. In this study, the uranium contaminants were decontaminated from the surface when the dried strippable polymer film was peeled off.

**Figure 2:**
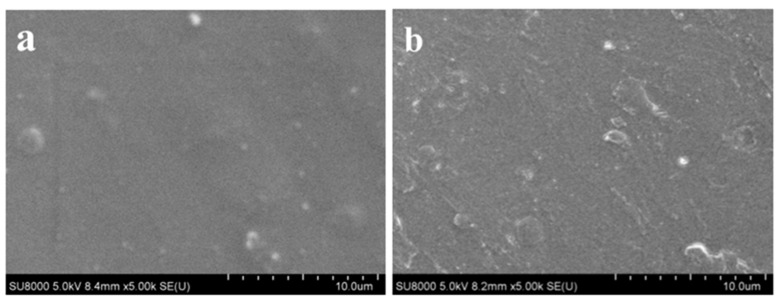
Micro morphology of dried polymeric film (**a**) C1 and (**b**) DeconGel 1101.

To assess the surface micro-morphology of dried polymeric film, the prepared C1 and commercial DeconGel 1101 samples were characterized by SEM and are shown in Figure 2. SEM images show the that stainless-steel plates are both uniformly covered by the two polymer coating films except for some folded protrusion of the DeconGel 1101 film. The smoothing surface of sample C1 could be attributable to its good filler dispersity and film formation, which increase decontamination performance. Figure 2 shows that there are obvious defects on the surface of the DeconGel 1101 film compared with that of sample C1; moreover, the surface of the DeconGel 1101 film was rougher than that of sample C1 at the same thickness (1 μm).

### 3.2. Surface Decontamination of Polychlorinated Biphenyls (PCBs) of the Polymer Strippable Coating

**Figure 3:**
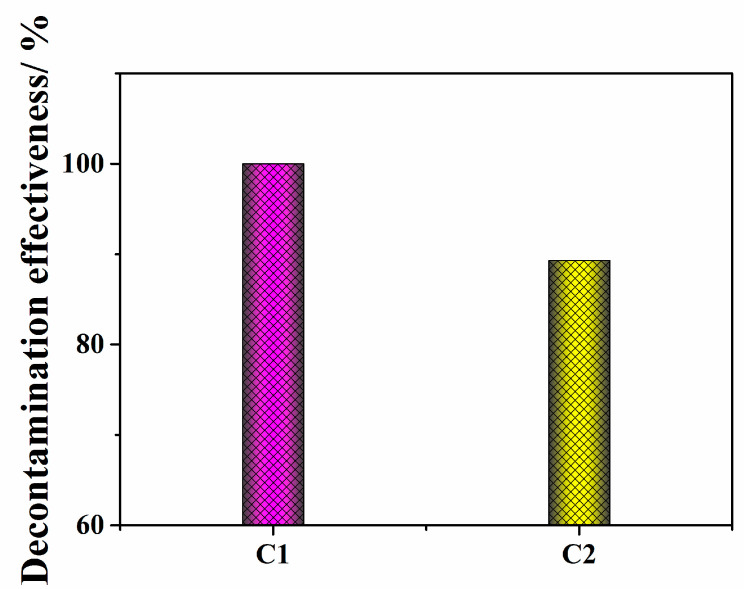
Decontamination effectiveness of PCBs detected by GC/MS.

PCBs, which have undesirable carcinogenicity, are chemically inert pollutants that persistently degrade the environment. The USS Missouri Maritime Museum vessel contains several areas with limited access to the public due to PCB oil contamination. In this study, PCBs were chosen as a marker to evaluate the primary decontamination performance of the strippable coating. Figure 3 shows the PCB decontamination effectiveness detected using GC/MS for the two prepared samples, and Figure S1 shows the PCB standard and TIC curves detected using GC/MS; C1 showed a 100.0% decontamination, in comparison with 89.3% for C2. Additionally, poured DeconGel 1101 showed a 92.4% decontamination on aluminum [33]. Table S1 lists the detailed PCB decontamination data detected using GC/MS. C1′s active components encapsulated the PCBs, which led to a excellent surface decontamination. There are numerous hydroxyl functional groups in the polymer chain of PVA and chitosan, which enabled hydrogen bonds to form between these groups and organic pollutants. More importantly, the amino groups on chitosan as an electron donor and the aromatic ring on PCBs as an electron acceptor interact with each other [34], differing in the absence of amino groups on starch, resulting in C1’s better decontamination. Based on these results, the formula for C1 is expected to be beneficial for the removal of uranium contaminants.

### 3.3. Performance and Stability of Surface Decontamination of Uranium Compounds for the Polymer Strippable Coating

**Figure 4:**
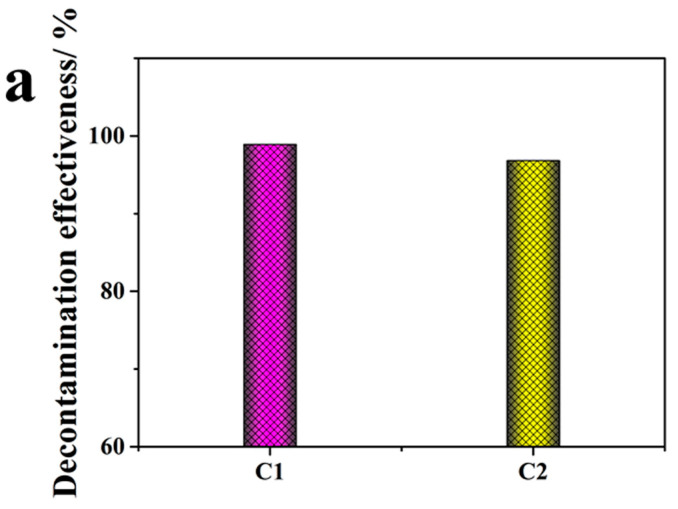

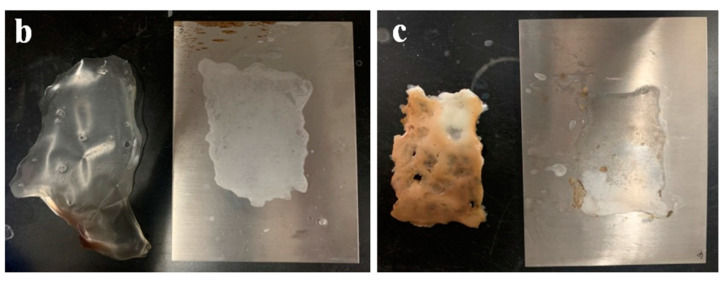
(**a**) Decontamination effectiveness and photographs of polymeric coating (**b**) C1 and (**c**) C2 for stainless steel plates.

To gain a better understanding of the decontamination, Table 2 presents all the decontamination values, and Figure 4 graphically displays the efficiencies for C1 and C2 for in a more accurate comparison. Both samples show a high efficiency for U decontamination from stainless steel plate surfaces, 98.9 ± 0.1% (C1) and 96.8 ± 0.7% (C2). The performance is higher than the reported values of between 80 and 96% using polyaniline doped with a SO_4_^2−^/TiO_2_ coating [24] and only about 23% for a commercial Alara strippable coating [5]. Sample C1 also showed a much greater decontamination effectiveness than the commercial DeconGel 1101 sample, with a rate of only 70.2% (shown in Figure S2) using the same decontamination test. The reasons could be that the uranium contaminants chelated with hydroxyl functional groups and fixed on the surface of the adsorbents and that the powerful chemical interaction between the U contaminant and the complexing agent increased the decontamination effectiveness. Furthermore, Figure 4a of C1 after it was peeled from the stainless-steel plates of C1 shows better film formation and peeling with no wrinkles or cavities, which meets the requirements for a decontaminant’s overall film formation, pollutant enrichment, and removal effect. The combination in C1 of CaCO_3_, EDTA-2Na, and modified chitosan embedded in PVA promoted the interpenetration of nanoparticles and film formation [28].

**Figure 5:**
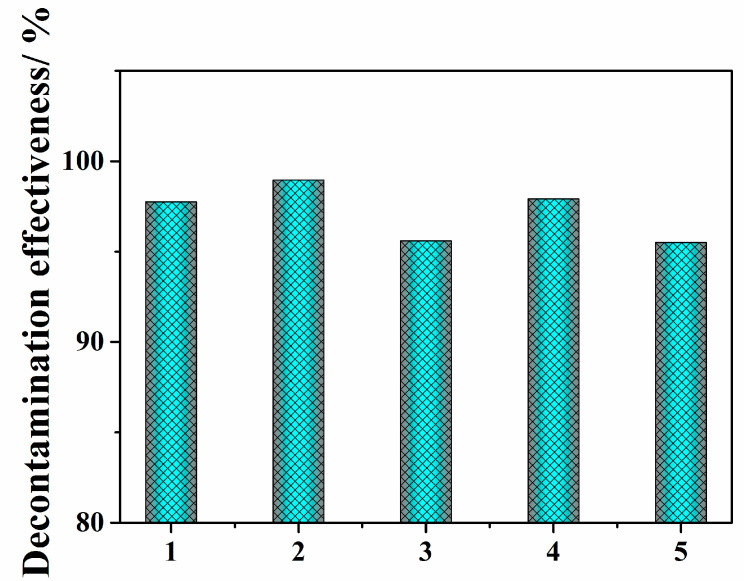
Repeatability of polymeric coating C1 (1 g/L) decontamination.

To evaluate the stability of the strippable coating for practical application, polymeric coating C1 (1 g/L) was applied 5 separate times as shown in Figure 5, and Table S2 presents the detailed data on C1’s decontamination repeatability. The table shows that decontamination ranged from 95.5 to 99.0%, which shows good repeatability and stability. Thus, it is anticipated that polymeric coating films composed of adsorbent and PVA possess good repeatable properties that make them suitable for large-scale industrial surface decontamination applications.

### 3.4. Comparison of Surface Decontamination of High Uranium Levels for the Polymer Strippable Coating

**Figure 6:**
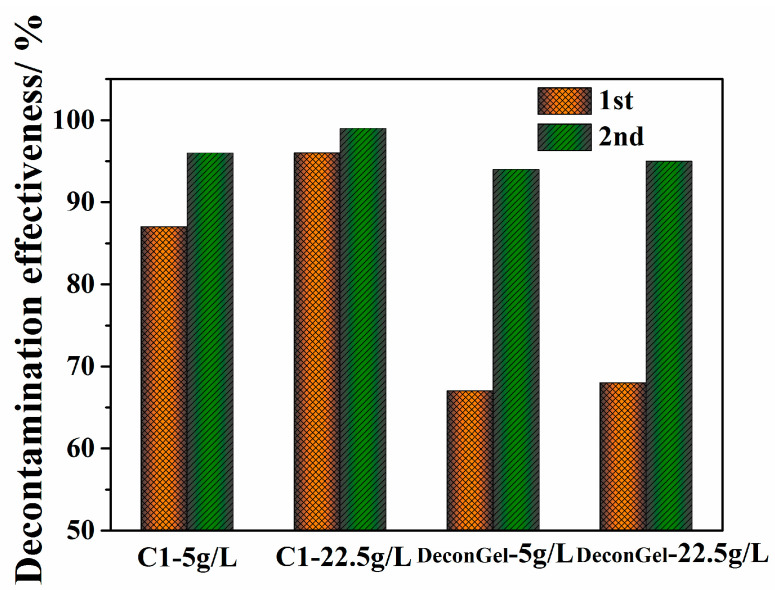
High decontamination effectiveness of polymeric coatings C1 and DeconGel 11001 with high surface contamination levels.

**Figure 7:**
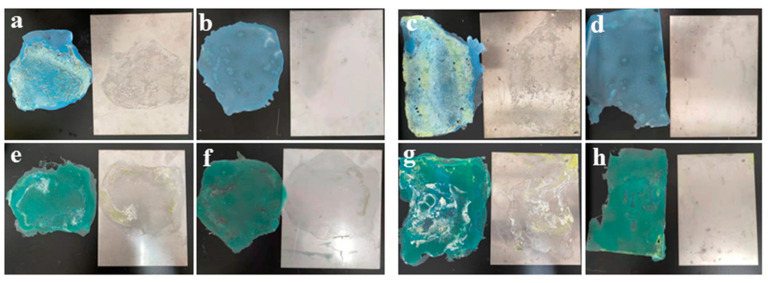
Photographs of high surface contamination levels: (**a**,**b**) 1st and 2nd CL decontamination of 5 g/L; (**c**,**d**) 1st and 2nd C1 decontamination of 22.5 g/L; (**e**,**f**) 1st and 2nd DeconGel decontamination of 5 g/L; (**g**,**h**) 1st and 2nd DeconGel decontamination of 22.5 g/L.

The high concentrations of surface contaminants vary greatly in practical U contamination facilities and can require multiple applications of a decontaminant to reduce the contaminants to acceptable levels. Therefore, a coating with a high decontamination effectiveness for both low and high U radiation levels is urgently needed. To measure their decontamination effectiveness, a decontamination test was performed with the prepared C1 sample and the commercial DeconGel 1101 and surface contamination concentrations of 5 g/L and 22.5 g/L (Figure 6); Table S3 shows the detailed data. At 5 g/L and 22.5 g/L of surface contaminant, sample C1 showed 87.2% and 95.5%, respectively, both considerably higher than those of commercial DeconGel 1101, only 66.7% and 68.5%, also respectively. On the second application, C1 showed a 95.5% and 99.3% decontamination of, respectively, 5 g/L and 22.5 g/L of surface contaminant; in comparison, DeconGel 1101 showed a decontamination of 94.1% and 94.7%, respectively. In all cases, the samples decontaminated more of the surface contaminant at the higher concentration. Gray reported that uranium decontamination decreased as the concentration of contaminant on the surface increased [5]; in that study, it is likely that the coating dried before it could attract more uranium. However, the greater decontamination effectiveness of C1 and DeconGel 1101 in this study could have been because the coatings achieved the full contact required for the larger amounts of contaminant to permeate the polymer. Figure 7 shows the high decontamination of both 5 g/L and 22.5 g/L of surface contaminant for both C1 and DeconGel 1101; in both decontaminations, both samples show an excellent film elasticity with both rigidity and easy removal from the surface (see Figure 7).

To determine the feasibility of applying the prepared adsorbent/PVA coating on vertical surfaces, C1 and DeconGel 1101 were applied to a vertical surface using the pouring method. It should be noted that both C1 and DeconGel 1101 samples are required to entirely coat the vertical surfaces, and this led to negligible inevitable flow with excellent anti-link and strippable properties. Therefore, the adsorbent/PVA polymeric coatings have excellent potential as new and cost-effective surface decontaminants that are suitable for the removal of high levels of surface U contaminants around nuclear facilities or following nuclear accidents.

### 3.5. Surface Decontamination of the Uranium Compounds for the Strippable Coatings on Various Substrates

**Figure 8:**
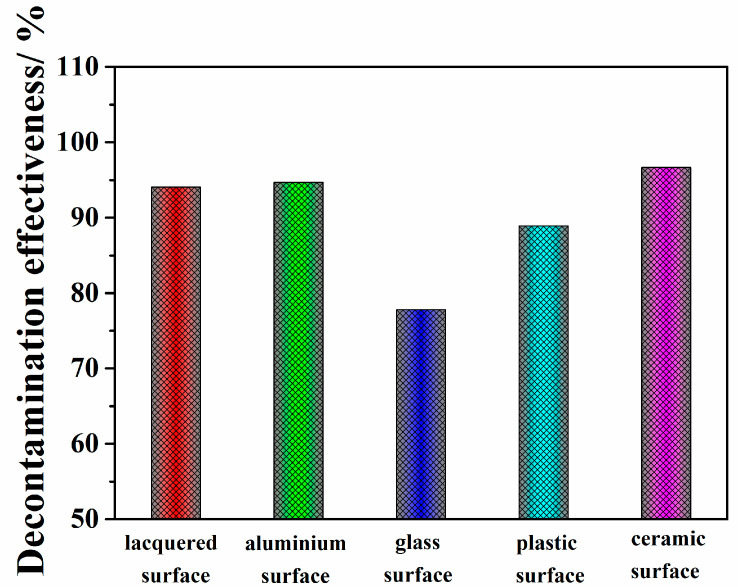
Decontamination effectiveness of polymeric coating C1 applied on different surfaces.

**Figure 9:**
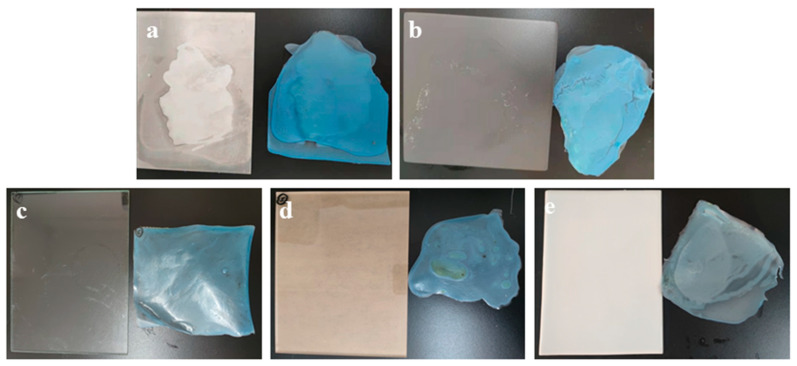
Photographs of decontamination of different surfaces: (**a**) lacquered surface, (**b**) aluminium surface, (**c**) glass surface, (**d**) plastic surface, (**e**) ceramic surface.

To evaluate the decontamination ability of the prepared adsorbent/PVA coating on rough and smooth surfaces, five U-contaminated lacquered, aluminum, glass, plastic and ceramic surfaces were chosen as representative models. Figure 8 shows high decontamination levels that varied from 77.8 to 96.7% for 1 g/L surface U in the sequence ceramic > aluminum > lacquered > plastic > glass. Table S4 presents the detailed C1 decontamination data for the different surfaces. Furthermore, the coatings all exhibit high adhesion and peeling off performances, as shown in Figure 9. The results indicate that the prepared coating has excellent potential for wide species surface decontamination after nuclear accidents or terrorist attacks.

### 3.6. Mechanical Performance of the Strippable Coating

**Figure 10:**
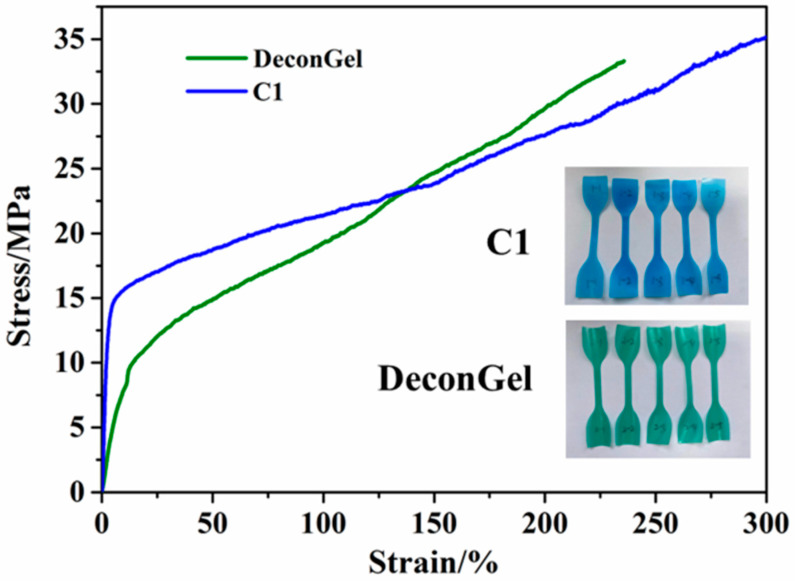
Stress–strain curves for polymeric coatings C1 and DeconGel.

Strength, flexibility, and ease of peeling off are necessary mechanical properties of an effective strippable coating film. Therefore, tensile tests were conducted with both C1 and DeconGel 1101 samples, as shown in Figure 10, and the tensile strength repeatability results are listed in Table S5. The stress–strain curves show that the tensile strength of C1 is 35.4 MPa, which is higher than that of DeconGel 1101. In addition, the elongation at the break of C1 is 302%, whereas that of DeconGel is 236%, indicating the greater flexibility of C1. These mechanical test findings indicate that both the tensile strength and elongation of C1 were higher than those of DeconGel 1101, which means that strippable coating C1 can be easily peeled off from the decontaminated surfaces without cracking. This good mechanical property can be attributed to the strength of modified chitosan and the great dispersibility of CaCO_3_. The PVA98+glycerol+EDTA+bentonite decontamination film is reported to display high elastic modulus [13].

## 4. Conclusions

In this study, a PVA-based strippable coating-containing adsorbent was successfully developed for the decontamination of U-contaminated surfaces. The adsorbents contained both the chelating agent EDTA-2Na and the nano-adsorbant CaCO_3_/TiO_2_. The proposed coating with a good repeatability and tensile strength showed a better surface U removal than the commercial DeconGel 1101 strippable coating due to the synergistic effect of employing the EDTA-2Na and CaCO_3_ fillers. Especially at high U contamination levels, the prepared PVA-based coating showed a high primary decontamination efficiency on the stainless-steel surface. Furthermore, the strippable coating also removed U contamination on both the horizontal and vertical surfaces of different substrates. Therefore, this coating represents a convenient, ecofriendly, and cost-effective option for U decontamination. It can be concluded that the adsorbent/PVA-based coating designed in this study has excellent potential as a new surface decontaminant with widespread application prospects in various nuclear fields.

## Figures and Tables

**Table 1:** Recipe for two aqueous polymeric coatings.

Samples	C1	C2
component	PVA	PVA
	CaCO_3_	TiO_2_
	modified chitosan	modified starch
	EDTA-2Na	EDTA-2Na

**Table 2:** Decontamination properties of two polymeric coatings.

Sample	Contaminant Level before Decontamination/Bq cm^−1^	Contaminant Level after Decontamination/Bq cm^−1^	Decontamination Effectiveness/%
C1	0.93	0.01	98.9 ± 0.1
C2	0.95	0.03	96.8 ± 0.7

## Data Availability

The data presented in this study are available on request from the corresponding author.

## Supplementary Materials

The following supporting information can be downloaded at: https://www.mdpi.com/article/10.3390/polym14091656/s1, Figure S1: PCB standard curve and TIC curves of PCB detected by GC/MS; Figure S2: Decontamination efficiency of polymeric coating C1 and Decongel 1101; Figure S3: Photographs of decontamination for vertically placed: (a,b) C1 and (c,d) decongel; Table S1: Decontamination efficiency of PCBs detected by GC/MS; Table S2: Repeatability of decontamination efficiency of polymeric coating C1 (1 g/L); Table S3: Decontamination properties of C1 and decongel 1101 for high unanium level of 5 g/L and 25 g/L; Table S4: Decontamination efficiency of polymeric coating C1 applied on different surfaces; Table S5: Repeatability of tensile strength tests for polymeric coating C1 and DeconGel.
