# Supplementary material for: Aqueous Strippable Polymer Coating for Highly Efficient Primary Radioactive Uranium Decontamination with Versatility on Diversified Surface
Source: Polymers (Basel). 2022 Apr 20;14(9):1656. doi: 10.3390/polym14091656 (PMC9100341; doi:10.3390/polym14091656)
Supplement: Supplementary file 1 [file polymers-14-01656-s001.zip › polymers-1661514-supplementary.pdf]

polymers

Article

Aqueous Strippable Polymer Coating for Highly Efficient Primary Radioactive Uranium Decontamination with Versatility on Diversified Surface

Yang Xue [1], Wuxinchen Yang [1,2], Renliang Yue [1] and Yunfa Chen [1,2,3,*,†]

[1] State Key Laboratory of Multi-Phase Complex Systems, Institute of Process Engineering, Chinese Academy of Sciences, Beijing 100190, China; yxue@ipe.ac.cn (Y.X.); ywxc666@163.com (W.Y.); rlyue@ipe.ac.cn (R.Y.)
[2] Center of Materials Science and Optoelectronics Engineering, University of Chinese Academy of Sciences, Beijing 100049, China
[3] Center for Excellence in Regional Atmospheric Environment, Institute of Urban Environment, Chinese Academy of Sciences, Xiamen 361021, China
* Correspondence: chenyf@ipe.ac.cn; Tel.: +86-10-82544894; Fax: +86-10-82544919
† Address: No.1 Zhongguancun Beiertiao, Haidian District, Beijing 100190, China.

Figures and tables

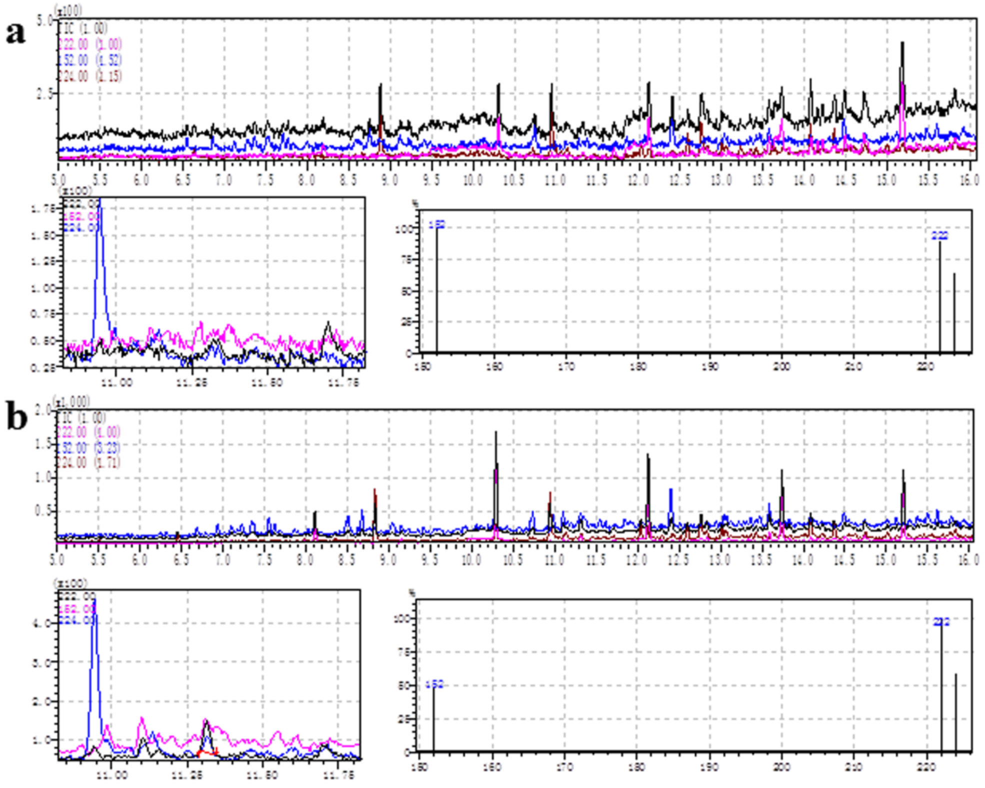

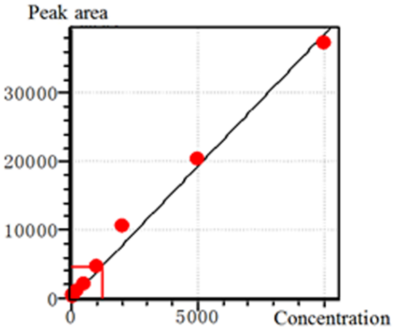

Figure S1. PCB standard curve and TIC curves of PCB detected by GC/MS.

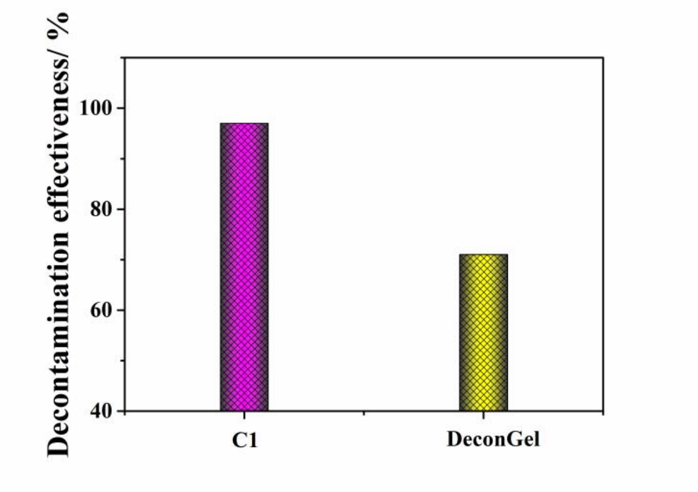

Figure S2. Decontamination effectiveness of polymeric coating C1 and Decongel 1101

Table S1. Decontamination effectiveness of PCBs detected by GC/MS

Sample	Contaminated level before decontamination/ppb	Contaminated level after decontamination/ppb	Decontamination Efficacy/%
	contaminated level	contaminated level	
C1	1208.81	0.00	100.0
C2	1208.81	131.34	89.3

Table S2. Repeatability of decontamination efficiency of polymeric coating C1 (1 g/L).

Sample	Contaminated level before decontamination/Bq cm^{-2}	Contaminated level after decontamination/Bq cm^{-2}	Decontamination Efficacy/%
	contaminated level	contaminated level	
1	0.89	0.02	97.8
2	0.97	0.01	99.0
3	0.91	0.04	95.6
4	0.96	0.02	97.9
5	0.89	0.04	95.5

Table S3. Decontamination properties of C1 and decongel 1101 for high unanium level of 5 g/L and 25 g/L.

Sample	concentration of unanium/g/L	Contaminated level before decontamination/Bq cm^{-2}	Contaminated level after decontamination/Bq cm^{-2}	Decontamination Efficacy/%
		contaminated level	contaminated level	
C1	5	4.70	0.60	87.2±0.4
-1st	22.5	12.05	0.54	95.5±0.3
Decongel	5	4.41	1.47	66.7±0.6
-1st	22.5	14.20	4.47	68.5±0.6
C1	5	4.70	0.21	95.5±1.7
-2nd	22.5	12.05	0.09	99.3±0.1
Decongel	5	4.41	0.26	94.1±0.3
-2nd	22.5	14.20	0.75	94.7±0.1

Table S4. Decontamination efficiency of polymeric coating C1 applied on different surfaces.

Surface	Contaminated level before decontamination/Bq cm^{-2}	Contaminated level after decontamination/Bq cm^{-2}	Decontamination Efficacy/%
	contaminated level	contaminated level	
ceramic	0.60	0.02	96.7±1.6
aluminum	0.94	0.05	94.7±1.2
lacquered	1.01	0.06	94.1±1.3
glass	0.72	0.16	77.8±1.5
plasic	0.63	0.07	88.9±1.3

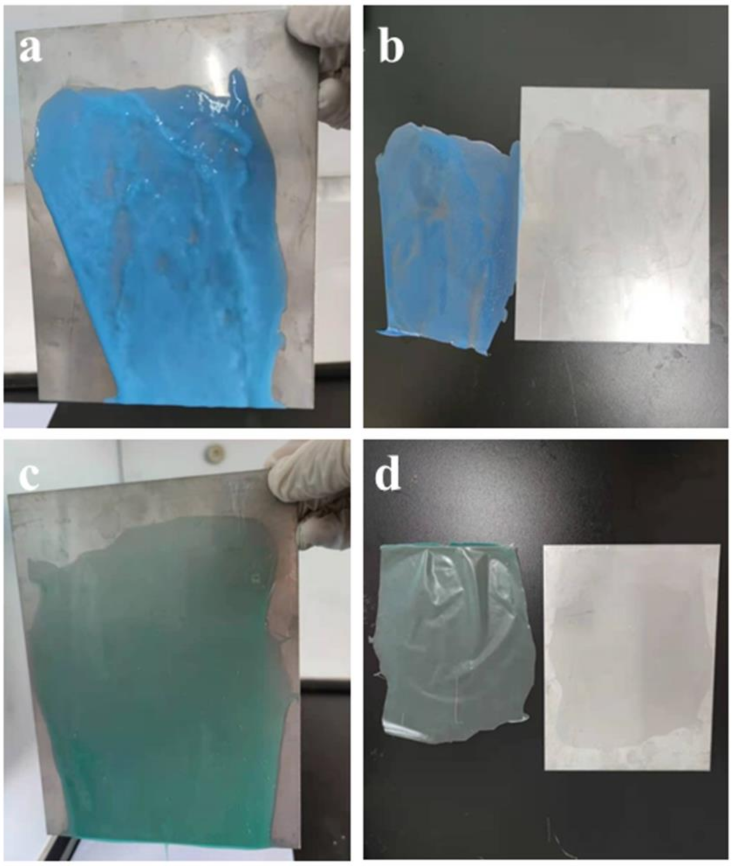

Figure S3. Photographs of decontamination for vertically placed: (a) (b) C1 and (c) (d) decongel.

Table S5. Repeatability of tensile strength tests for polymeric coating C1 and DeconGel.

Sample	Tensile strength/Mpa		Elongation at break/%	
	C1	Decongel	C1	Decongel
1	36.4	33.3	329	236
2	36.8	32.7	339	237
3	35.1	34.3	299	229
4	35.4	28.1	302	164
5	34.9	33.6	308	234
